# PlasCAT: Plasmid Cloud Assembly Tool

**DOI:** 10.1093/bioinformatics/btae299

**Published:** 2024-05-02

**Authors:** Samuel Peccoud, Casey-Tyler Berezin, Sarah I Hernandez, Jean Peccoud

**Affiliations:** GenoFAB, Inc., Fort Collins, CO 80528, United States; Department of Chemical and Biological Engineering, Colorado State University, Fort Collins, CO 80523, United States; Department of Chemical and Biological Engineering, Colorado State University, Fort Collins, CO 80523, United States; GenoFAB, Inc., Fort Collins, CO 80528, United States; Department of Chemical and Biological Engineering, Colorado State University, Fort Collins, CO 80523, United States

## Abstract

**Summary:**

PlasCAT (Plasmid Cloud Assembly Tool) is an easy-to-use cloud-based bioinformatics tool that enables *de novo* plasmid sequence assembly from raw sequencing data. Nontechnical users can now assemble sequences from long reads and short reads without ever touching a line of code. PlasCAT uses high-performance computing servers to reduce run times on assemblies and deliver results faster.

**Availability and implementation:**

PlasCAT is freely available on the web at https://sequencing.genofab.com. The assembly pipeline source code and server code are available for download at https://bitbucket.org/genofabinc/workspace/projects/PLASCAT. Click the Cancel button to access the source code without authenticating. Web servers implemented in React.js and Python, with all major browsers supported.

## 1 Introduction

Synthetic DNA constructs such as plasmids play a major role in the emerging bioeconomy, yet the verification of DNA sequences is often overlooked (Peccoud *et al.* 2011, Thuronyi *et al.* 2023). Sequences can be verified through reference-based assembly of sequencing reads; however, it is often necessary to perform *de novo* assembly of DNA sequences because there is no reference sequence available or to avoid reference bias in detecting variants (Peccoud *et al.* 2011, Gallegos *et al.* 2020, Chen *et al.* 2021, Valiente-Mullor *et al.* 2021). Much attention has been paid to *de novo* genome assembly (De Maio *et al.* 2019, Boostrom *et al.* 2022, Khrenova *et al.* 2022). Unfortunately, the tools designed for genome assembly often misassemble, or completely miss, small (∼10 kb) plasmids (Johnson *et al.* 2023). A few tools have sought to uncover plasmids from whole genome or metagenome datasets (Antipov *et al.* 2016, Antipov *et al.* 2019, Gomi *et al.* 2021, Pellow *et al.* 2021, Rozov *et al.* 2017), but only two tools, to our knowledge, are designed only for *de novo* plasmid assembly. One was developed by Oxford Nanopore Technologies (ONT) for use with long-reads (https://github.com/epi2me-labs/wf-clone-validation), while the other was developed only for short-reads (Gallegos *et al.* 2020). Furthermore, the need for assemblies with base-pair precision is much greater with these small plasmids than with large genomes, since plasmids are often used to produce clinical products, such as recombinant insulin or vaccines, or to design genetic circuits (Brophy and Voigt 2014, Martínez-Puente *et al.* 2022).

Although short sequencing reads from Illumina have high accuracy, the fragmented nature of the library as well as sequencing biases introduced during library preparation and/or PCR can make it difficult to produce a complete assembly or to resolve repetitive regions (Liao *et al.* 2019). Conversely, long sequencing reads have historically been marred by high error rates, although their length can help resolve complex or repetitive sequences (Liao *et al.* 2019). Yet even as the accuracy of the Nanopore chemistry and basecalling methods improve, long-reads are still prone to introducing insertions and/or deletions (indels) in the final assembly (Xia *et al.* 2023). As such, a hybrid approach to sequencing assembly, utilizing both short-reads and long-reads, is commonly recommended and will likely improve plasmid assemblies (De Maio *et al.* 2019, Johnson *et al.* 2023). Unicycler is the current gold standard hybrid *de novo* assembly tool (Wick *et al.* 2017, De Maio *et al.* 2019, Johnson *et al.* 2023).

Here, we improve the *de novo* plasmid assembly pipeline described by (Gallegos *et al.* 2020) in two major ways: (i) assemblies can now be generated from Illumina short-reads, Nanopore long-reads, or a hybrid approach utilizing both short- and long-reads; and (ii) the pipeline is implemented onto an easy-to-use, cloud-based web application. Even with no prior computational experience, individuals can use PlasCAT (Plasmid Cloud Assembly Tool) to generate reliable *de novo* plasmid assemblies directly from raw sequencing data.

## 2 Implementation

**Figure 1.**
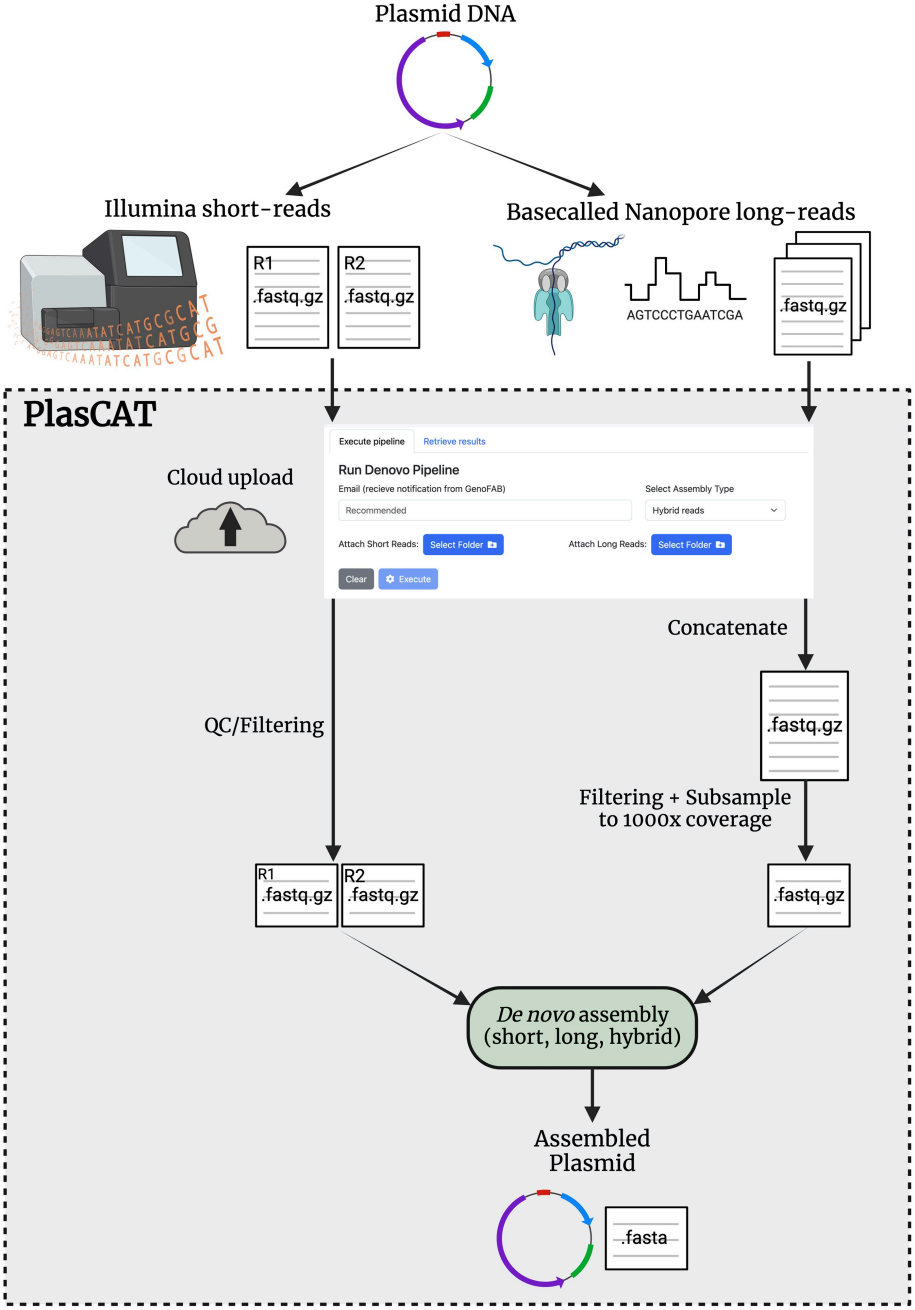
PlasCAT workflow. Upon sequencing plasmid DNA with Illumina short-read and/or Nanopore long-read technologies, raw (basecalled) data are uploaded to the cloud via a simple web interface. Sequencing read files are processed and used for *de novo* assembly. The final result is a FASTA file containing the plasmid sequence.

The PlasCAT workflow is represented in Fig. 1. The user interface or front-end of PlasCAT is written in React.js and hosted on Elastic Compute Cloud (EC2), a product of Amazon Web Services (AWS). The back-end, which performs the sequence assembly and verification, is hosted on a separate EC2 instance with greater computing resources. The back-end uses Flask, a Python micro web framework, to implement a Representational State Transfer Application Programming Interface (REST API). The sequencing files that are processed by the back-end are first uploaded from the React app to an AWS Simple Storage Service (S3) bucket. Depending on the amount of sequencing data uploaded, the sequence assembly and verification pipeline can take longer than 60 s—the typical API timeout duration. To prevent timeouts, the REST API starts a background process on the web server using Celery, an asynchronous task queue service. A Celery process is created for each API call that executes the pipeline. The Celery process first copies all the files from the S3 bucket to the web server, and then each plasmid sequence is assembled on a separate thread of the server’s CPU using Python’s multiprocessing package. After the files are uploaded, the user receives a unique process ID which is used to retrieve the final output.

The assembly pipeline is run from a Docker container on the server. Thus, users do not have to maintain package installations on their own system nor invest in equipment with high computing power, saving time, money and increasing reproducibility. Long-read FASTQ files corresponding to the same sample are first concatenated into one file. Filtering of long-reads is then performed with Filtlong (https://github.com/rrwick/Filtlong), where the worst 20% of reads and any reads longer than 20 000 bp are removed. The long-read data is then randomly subset to 1000x coverage using rasusa (Hall 2022). For this, the user must provide an estimated size for the plasmid. It has been shown that subsetting sequencing data cannot only reduce processing time but also improve assembly results, even more so than filtering alone, and has been implemented in many assembly tools (Lonardi *et al.* 2015, De Maio *et al.* 2019, Wick and Holt 2019, Murigneux *et al.* 2020, Bouras *et al.* 2023). Indeed, assembly quality can plateau as sequencing depth increases (Zhang *et al.* 2023). Following this pre-processing of long-reads, the pipeline proceeds as previously described (Gallegos *et al.* 2020). In brief, short reads are first filtered by quality and length through Trimmomatic (Bolger *et al.* 2014). Then, the assembly is performed by passing the forward and reverse short reads and/or long reads to Unicycler (Wick *et al.* 2017). Typically, a single circular contig representing the plasmid will be generated; however, depending on the dataset, multiple contigs may be output. This may represent either a true mix of DNA sequences in the sample or indicate inadequate data for assembly generation.

If there is a failure in a long-read or hybrid assembly, the assembly process will automatically re-run, up to three times, using new seeds for rasusa subsampling. After all the sequence assemblies complete or fail, the output files are uploaded to the S3 bucket. If the user opts to enter their email address, they automatically receive an email with a zip file containing a folder for each plasmid with the sequence assembly file (.fasta), Unicycler log file, and a Graphical Fragment Assembly file (.gfa). A csv file is also produced that summarizes the output of the assembly pipeline for each plasmid, i.e. whether the assembly succeeded, the number and length of the contigs, and the duration of each assembly process. The summary information is also displayed at the bottom of the notification email. If the user does not enter their email, they can alternatively download the same zip file by entering their issued process ID on the front-end after the process finishes.

## 3 Usage

To start an assembly, users fill out fields and provide data on their sequencing reads in the “Execute Pipeline” tab. First, users may provide their email addresses to receive the resulting assemblies via email. Then, users select the appropriate assembly method for their data: short-read, long-read, or hybrid using both short and long reads. The pipeline takes as input compressed FASTQ files (.fastq.gz) containing the raw paired-end Illumina short-reads and/or compressed FASTQ files containing basecalled Nanopore long-reads. Users have no pre-processing to do between getting their data off the sequencing machine and uploading it to the web app. Example FASTQ files for testing PlasCAT are available on the website and in the source repositories.

The application expects the reads for each plasmid to be in their own directories, consistent with how data are output from the sequencers. Short-read data files must have a name containing either “R1” or “R2” to signify whether the reads are the forward or reverse reads, respectively, and the folder’s name containing these two files will determine the plasmid’s name. Long-read data files can be supplied as individual basecalled files or as concatenated files. The sample name will be automatically determined from the folder name. Once a folder containing short- or long-reads is selected, a table will appear below the form showing the sample information. Additional folders can be added by repeating the process. For short-read assembly, users should verify that the information in the table looks correct and then press execute to start the assembly. For assemblies from long reads, an estimate for the size of the DNA molecule (in base pairs, bp) must be entered in the table to subset the reads to a particular coverage. For hybrid assembly, users must ensure that the long reads and short reads are accurately mapped to each other by placing them in the same row. The short read list can be reordered by dragging the row up and down the short read table. It is recommended to test a single plasmid assembly before running all the assemblies.

Once the “Execute” button is pressed, the application will upload all the files to the cloud, and a green checkmark will flash on the screen indicating that the process has started. A process ID will be issued and displayed on the page which the user should save. While the app uploads the files, the user must keep the tab open, but after the uploading indicator goes away and the checkmark flashes, the tab can be closed. Depending on the amount of data in a process and the traffic on the website, the pipeline can take tens of minutes to produce assemblies. If the user entered their email, they will be notified when the process is complete. The user can check the status of the process by clicking “Retrieve results,” and entering the process ID. If it is still running, a notification appears stating “Assembly still in progress…” and the page can be automatically refreshed every 10 s until the assemblies finish. A good rule of thumb to estimate the time for a process to complete is about five minutes per assembly, with long-read assembly generally faster than short-read or hybrid assembly. Once the assembly is finished, the page will stop refreshing and the results will be automatically downloaded. The data related to a process is guaranteed to be stored for at least 24 h after the process finishes. Users can assess the quality of the final assemblies by viewing the gfa assembly file with a tool like Bandage (Wick *et al.* 2015), or the FASTA sequence file with a tool like SnapGene.

## 4 Discussion

PlasCAT enhances the accessibility and speed of plasmid assembly while enforcing data format restrictions to ensure robust assembly. This tool represents a shift in the way scientists interact with bioinformatics pipelines, moving from tools for the coding-savvy to a simple step in their lab's workflows. In contrast to many open-source software options, dedicated support for the assembly tool is available through GenoFAB, Inc. New assembly techniques and parameters can be easily added to the platform through updates to the pipeline without any change in user experience. This is critical given *de novo* and plasmid-centric assembly techniques will continue to improve. Large libraries of plasmids are needed to assess the quality of different processing and assembly tools, thus future work will generate many plasmid samples for a thorough benchmarking of assembly tools; superior tools can be quickly integrated into the PlasCAT pipeline. Currently, the hybrid approach to *de novo* plasmid assembly with PlasCAT is more robust than the short-read and long-read only approaches, in terms of both assembly success and reproducibility across repeated library preparations (Hernandez *et al.* 2024). In addition, the long-read assemblies were similar to those obtained from ONT’s Epi2ME tool, although neither performed as well as the hybrid assemblies (Hernandez *et al.* 2024).

The ability for nontechnical users to quickly perform different types of plasmid assemblies, especially a hybrid approach that has shown promise for identifying plasmids in genomic datasets, represents an important advancement. Despite its improvements to the assembly process, PlasCAT has a few limitations, primarily related to assembly size and the nature of the tool. The tool is designed to assemble small plasmids smaller than 20 000 bp, making the current version unsuitable for larger genome assembly. Additionally, assembling many plasmids simultaneously can result in longer upload times compared to local assembly, where there is no upload needed. However, powerful computer servers with multiprocessing can speed up the assembly process. Lastly, because PlasCAT is fully cloud-based, there are increased security concerns regarding data privacy for uploaded content. This makes PlasCAT less applicable for sequences under strict security standards, such as HIPAA. Using the open-source pipeline for local assembly is a more suitable approach for sensitive data, while still allowing users to modify parameter values set by the pipeline as needed (e.g. maximum or minimum read size for filtering).
